# Causal associations between gut microbiota and synovitis–tenosynovitis: a two-sample Mendelian randomization study

**DOI:** 10.3389/fmicb.2024.1355725

**Published:** 2024-04-30

**Authors:** Xietian Yin, Shichao Zhao, Zhangkui Tan, Jun Xu, Qiping Lu

**Affiliations:** ^1^Post-Doctoral Research Workstation, General Hospital of Central Theater Command of the PLA, Wuhan, China; ^2^College of the First Clinical, Hubei University of Chinese Medicine, Wuhan, China; ^3^Department of Rheumatology, Hubei Provincial Hospital of TCM, Wuhan, China; ^4^Department of Geriatrics, Hubei Provincial Hospital of TCM, Wuhan, China; ^5^College of Basic Medicine, Hubei University of Chinese Medicine, Wuhan, China

**Keywords:** Mendelian randomization, causality, gut microbiota, synovitis, tenosynovitis

## Abstract

**Background:**

Increasing evidence indicates that gut microbiota dysbiosis is related to synovitis and tenosynovitis. Nonetheless, whether these associations are causal is currently unknown.

**Objectives:**

A two-sample Mendelian randomization (MR) study was performed to reveal the causality of gut microbiota with synovitis and tenosynovitis.

**Methods:**

The summary statistical data from a large-scale genome-wide association study (GWAS) were applied as the basis for a two-sample MR analysis. The causal effect was estimated using inverse variance weighted (IVW), weighted median, simple mode, MR-Egger, and weighted mode methods, of which IVW was the important method. Meanwhile, the pleiotropy and heterogeneity were detected and measured using MR-Egger regression, Cochran’s Q statistics, funnel plots, and MR pleiotropy residual sum and outlier (MR-PRESSO) methods.

**Results:**

The IVW technique demonstrated that genetically predicted five genera, namely *Gordonibacter* [odds ratio (OR) = 0.999, 95% confidence interval (CI): (0.9977, 0.9998), *p* = 0.019], *Paraprevotella* [OR = 0.999, 95% CI: (0.9971, 0.9999), *p* = 0.036], *Lachnoclostridium* [OR = 0.998, 95% CI: (0.9954, 0.9999), *p* = 0.041], *RuminococcaceaeUCG003* [OR = 0.997, 95% CI: (0.9955, 0.9994), *p* = 0.011], and *FamilyXIIIAD3011group* [OR = 0.997, 95% CI: (0.9954, 0.9992), *p* = 0.006] were negatively correlated with the risk of synovitis and tenosynovitis, while two other genera, namely *Ruminococcustorquesgroup* [OR = 1.003, 95% CI: (1.0004, 1.0049), *p* = 0.019] and *Parabacteroides* [OR = 1.003, 95% CI: (1.0002, 1.0052), *p* = 0.035] were positively associated with synovitis and tenosynovitis risk. In addition, the data of sensitivity analyses demonstrated that there were no outliers, horizontal pleiotropy, or heterogeneity in the causal relationship of the above-mentioned gut microbiota on synovitis and tenosynovitis (*p* > 0.05).

**Conclusion:**

The results of the study suggested that the gut microbiota was causally involved in synovitis and tenosynovitis and identified specific bacterial taxa that affect synovitis and tenosynovitis, which provide new insights into the pathogenesis underlying the development of synovitis and tenosynovitis mediated by gut microbiota.

## Introduction

Synovitis and tenosynovitis are a set of aseptic inflammatory diseases that relate to chronic strain or acute trauma and are also seen in diseases of the immune system. The incidence of synovitis and tenosynovitis is estimated to be very high in the general population, and research has shown that the incidence of synovitis in China was as high as 25%, with rates in Shanghai and Shenzhen reaching up to 35%. In 1996, a World Health Organization survey found that the incidence of synovitis in the United States was approximately 35%, in Britain and France, it was 25–30%, and in Japan, it was approximately 20%. Some studies have shown the incidence of tenosynovitis was 0.2–2.6%, and the trend is obviously increasing. Synovitis and tenosynovitis can occur at all ages, and their prevalence increases gradually with age, with female individuals being higher than male individuals. Moreover, the prevalence of synovitis and tenosynovitis varies among different occupational groups and is positively correlated with labor intensity (Maĭko et al., 2005; Guillén Astete et al., 2014; Bao et al., 2020). According to relevant information, the total prevalence of synovitis and tenosynovitis in Brazil in 2008 had reached 10.9/10000, which occurred predominantly in manual occupations and was a group of second most common musculoskeletal disease, after back disorders. Due to its high prevalence, it can seriously affect the work life and health of individuals and result in high medical costs to society. A 6-year follow-up study conducted in Brazil analyzed the risk factors of absenteeism among judiciary court employees owing to musculoskeletal diseases, which showed that the incidence of sick leave was 23%, with synovitis and tenosynovitis at 8.8%. A study of Brazilian Social Security data showed that musculoskeletal diseases accounted for 22.0% of cases of sickness benefits for formal contract employees. Among these, synovitis and tenosynovitis accounted for 11.6%, and if only female sickness beneficiaries with accidents were considered, synovitis and tenosynovitis were the most persistent and prevalent causes (Andrade and Barbosa-Branco, 2015; França et al., 2021; Guo et al., 2023; Sahbudin et al., 2023). Thus, the management and prevention of synovitis and tenosynovitis have been recognized by scholars as one of the serious public health problems that need to be addressed urgently.

Currently, the biggest known symbiotic microbiological communities in humans are the gut microbiota, and this symbiotic relationship plays an important role in human diseases and health (Shkoporov and Hill, 2019; Li et al., 2023). The gut microbiota affects the physiology of the host by participating in various biological processes, which include regulating immune function, regulating the oxidative stress response, maintaining anabolic balance, resisting inflammation and infection, and so on (Adak and Khan, 2019; Ticinesi et al., 2019; Chen et al., 2023b). An increasing number of studies have confirmed that gut microbiota dysbiosis is associated with synovitis and tenosynovitis susceptibility and progression. According to the study of Imoto et al., Mycobacterium virginiense could induce flexor digitorum profundus and superficialis muscles synovitis and tenosynovitis (Imoto et al., 2023). Additionally, based on recent research of Jiang et al., the prevalence of hand synovitis was related to gut microbiota dysbiosis (Jiang et al., 2023). It is generally believed that synovitis and tenosynovitis are more common in rheumatoid arthritis (RA) and osteoarthritis (OA), which are the main pathological manifestations of these two diseases (Liu et al., 2017; Hoque et al., 2023; Ishizaki, 2024). At present, a plethora of evidence has confirmed that gut microbiota dysbiosis is closely related to the occurrence and development of RA and OA and can further lead to synovitis and tenosynovitis, which is called the gut–joint axis (Xiang et al., 2022; Xu et al., 2022). Based on the research of Liang et al., fecal comparative analysis of RA patients and healthy people indicated that the contents of *Flavobacterium*, *Eisenbergia*, *Escherichia coli*, and *Klebsiella* in the feces of RA patients were higher, while the contents of *Enterococcus*, *Pseudomonas*, and *Fusobacterium* in the feces of healthy individuals were higher (Liang et al., 2023). According to some studies, fecal samples from RA patients were analyzed and showed an increase in *Wallemia* and *Candida* species abundance, while *Trichosporon*, *Scedosporium*, and *Pholiota* species abundance decreased, which further indicated that the difference in the fungal microbiome might contribute to the inflammatory reaction in RA patients (Dagar et al., 2022). According to other studies, *Lactobacilli* was related to the onset of OA, which also could be diagnosed and treated through *Lactobacilli* (Marchese et al., 2023). At the genus level, the abundance of *Candida*, *Cryptococcus*, and *Saccharomyces* was higher in OA patients (Jeyaraman et al., 2023). After analyzing fecal samples from OA patients and healthy individuals through deep whole-metagenome shotgun sequencing, it was found that the gut microbiome (encompassing virome, mycobiome, and bacteriome) of OA was radically altered compared to healthy people, encompassing 627 viral operational taxonomic units, 10 fungal species, and 279 differentially abundant bacterial species, indicating the gut microbiota has potential in predicting OA and related diseases (Chen et al., 2023a). Based on the close connection between synovitis and tenosynovitis with the aforementioned diseases, the appearance of synovitis and tenosynovitis should also be theoretically related to the imbalance of gut microbiota. Although prior studies have linked gut microbiota to synovitis and tenosynovitis, these findings are mainly case reports and observational studies, and the results are not very robust. At the same time, a large amount of research focused on musculoskeletal diseases, without systematically discussing the relationship between gut microbiota with synovitis and tenosynovitis. Therefore, it is currently unclear whether there is causality between them.

Mendelian randomization (MR) is a statistical approach that deduces causality between exposures (particular risk factors) and outcomes (specific phenotypes) by utilizing instrumental variables (IVs) (Birney, 2022). The IVs are genetic variants so that the MR study can avoid the effect of traditional confounders (Grover et al., 2017; Bowden and Holmes, 2019). Currently, due to the widespread application of MR analysis and the fruitful results of genome-wide association study (GWAS) on disease and gut microbiota levels, we used the single nucleotide polymorphisms (SNPs) from the GWAS (as IVs) for this two-sample MR study, in order to determine whether gut microbiota affect synovitis and tenosynovitis.

## Materials and methods

### Description of the MR research assumptions and design

**Figure 1:**
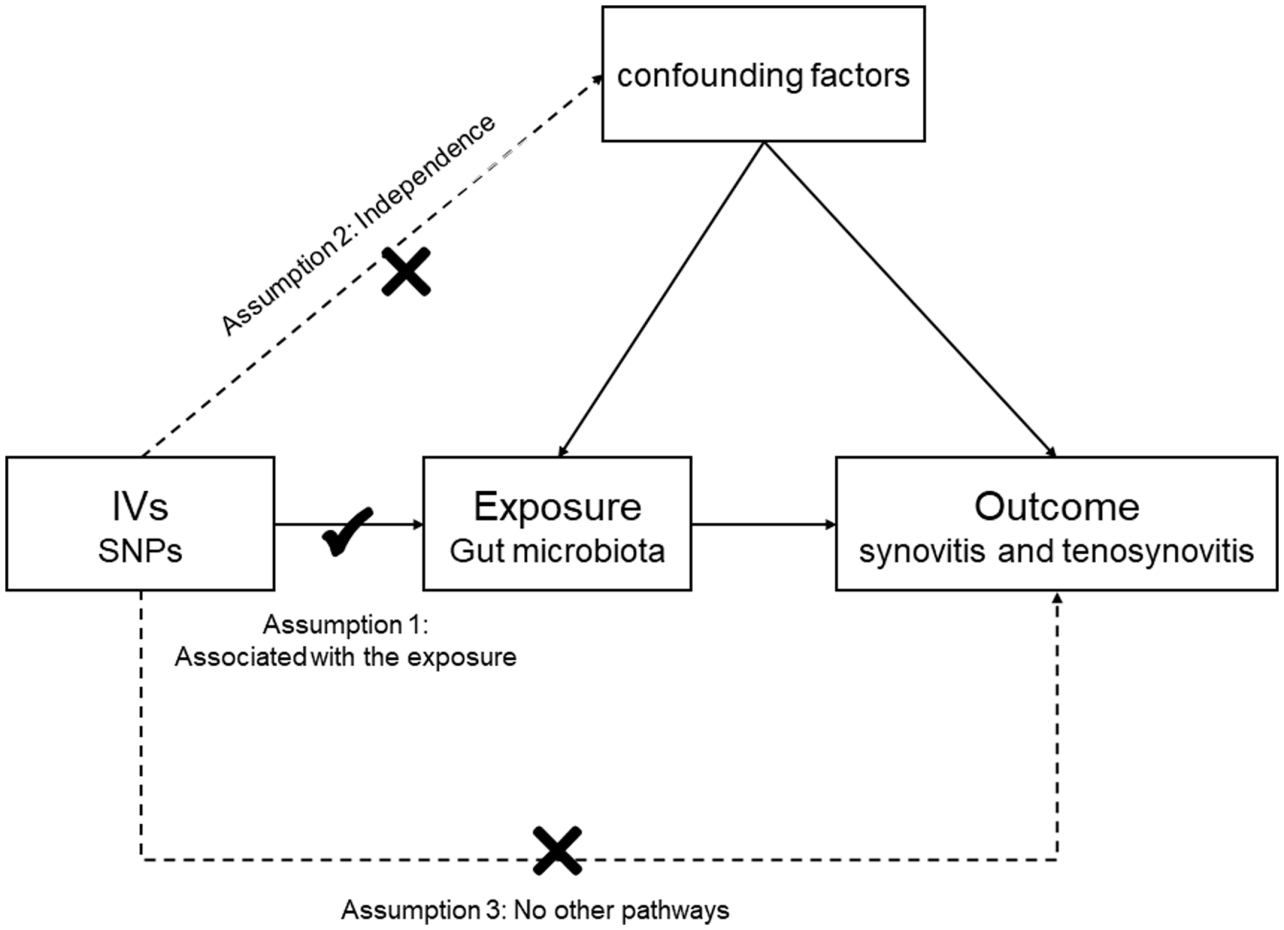
Three assumptions of the MR study.

**Figure 2:**
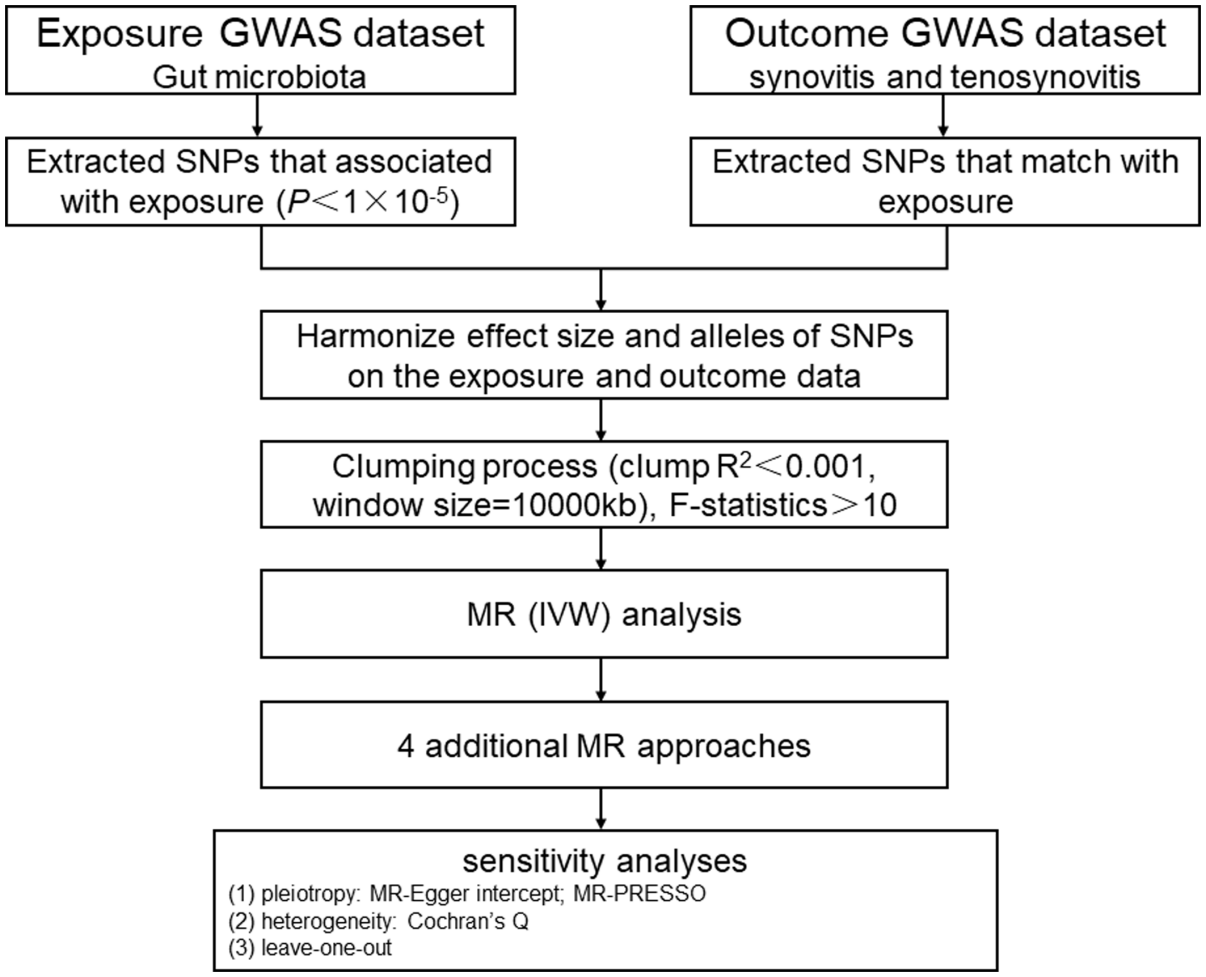
MR study flowchart. GWAS, genome-wide association study; SNPs, single nucleotide polymorphisms; MR, Mendelian randomization; IVW, inverse-variance weighted; MR-PRESSO, MR pleiotropy residual sum and outlier.

In this study, we chose gut microbiota as the exposure, with synovitis and tenosynovitis as the outcomes, and SNP loci as IVs. Based on this premise, we found that gut microbiota was significantly associated with synovitis and tenosynovitis, which was subsequently analyzed through an MR study. The MR research should insist on the following three assumptions (Figure 1): (1) associational hypothesis: genetic variants (as IVs) are robustly related to exposure; (2) independence hypothesis: genetic variants (as IVs) are not associated with confounders influencing exposure and outcome; (3) exclusivity hypothesis: genetic variants (as IVs) can only influence outcome risk through exposure, not the other way (Davies et al., 2018). We explored the causal influence of gut microbiota on synovitis and tenosynovitis performing a two-sample MR approach, which was undertaken following a flowchart as outlined in Figure 2. In this MR investigation, we conducted a comprehensive sensitivity analysis, including a test for pleiotropy, a test for heterogeneity, and a leave-one-out analysis. At the same time, we published the results according to the MR-STROBE recommendations (Skrivankova et al., 2021).

### Ethics statement

The data involved in this MR study were all collected from publicly available GWAS summary statistics, and there was no new data, therefore, a new ethical approval was not needed [data from^1^ and^2^].

### Data sources

Our GWAS summary data pertaining to gut microbiota were obtained from the MiBioGen consortium, which was committed to a better understanding of the gut microbiota genetic architecture. It conducted the biggest, multiracial, full-genome meta-analysis of gut microbiota up to now. It included 24 cohorts, with 18,340 participants, genotyped adopting whole-genome SNP arrays, while the gut microbiota was studied through 16S ribosomal RNA sequencing. Then, mapping analysis of microbiota quantitative trait loci (mbQTL) was used in search of host genes at genetic loci linked to the various gut microbiota abundance levels. After data processing, 211 taxonomic groups including genera 131, families 35, orders 20, classes 16, and phyla 9 were identified. Eventually, 12 unknown genera were excluded, and a total of 119 genera were included as the subjects of this research (Kurilshikov et al., 2021). Our GWAS summary data for synovitis and tenosynovitis were obtained from the publicly available GWAS analyses, with a total sample size of 361,194 European ancestry, encompassing 358,382 controls and 2,812 cases, for a total of 11,090,107 SNPs. Patients with synovitis and tenosynovitis are defined by ICD-10 code M65.

### Selection of variables

To ensure an accurate and effective conclusion of the causality of gut microbiota with synovitis and tenosynovitis, we adopted the quality control procedures below to filter suitable IVs: (1) The IVs were closely correlated with the corresponding exposures, and to ensure that the results obtained were more comprehensive, SNPs related to gut microbiota were selected as potential IVs at the locus-wide significance threshold (*p* < 1 × 10^−5^; Wang et al., 2023). (2) The IVs associated with exposure were independent of each other, and we used the European sample data as a reference panel (1,000 Genomes Project) to exclude the linkage disequilibrium (LD) between the SNPs to avoid the offset they caused (*r*^2^ < 0.001, LD distance >10,000 kb) (Cao et al., 2023). (3) *F*-statistic was used to measure the IVs and exposure relationship strength, with a higher *F*-statistic indicating the greater effect of IVs. *F*-statistic >10 was considered to be strong IVs and retained, while *F*-statistic ≤10 took into account to be weak IVs and ruled out. We used the following formula to calculate the *F*-statistic: *F* = (*n*-k-1) *R*^2^/k (1-*R*^2^), where *n* represents the sample size, *k* represents the number of IVs, and *R*^2^ represents the degree to which IVs explain exposure factors. Meanwhile, the specific calculation method for *R*^2^ was as follows: *R*^2^ = 2 (1-MAF) MAF *β*^2^, with MAF denoting the minor allele frequency, and *β* indicating the size of the effect of SNP on exposure factors (Au Yeung et al., 2018).

### Statistical analysis

We used several statistical models for MR analysis, encompassing simple mode, MR-Egger, inverse variance weighted (IVW), weighted median (WME), and weighted mode methods, to determine whether gut microbiota has a causal relationship with synovitis and tenosynovitis, with IVW being utilized as the primary analysis method. The IVW approach aggregated Wald values of each SNP and derived the effect overall estimates through meta-analysis. However, the IVW results would be seriously biased if horizontal pleiotropic SNPs were present (Burgess et al., 2017), so the other methods mentioned above could serve as additional complementary ways for evaluating causal effects in different situations. In the absence of pleiotropy and heterogeneity, the IVW estimation results are preferred. The MR-Egger approach could be used to evaluate the horizontal pleiotropy of genetic variants and could provide an estimation of the causal effect consistent with the IVW when the pleiotropic SNPs associated with the results were excluded (Bowden et al., 2015; Zheng et al., 2017). The MR-Egger estimation results are preferred when there is pleiotropy. The WME approach weighted the causal effect of different genetic variants on traits and then took its median as the final causal effect estimate. Even when 50% of the weight was derived from invalid IVs, it could provide consistent estimates (Bowden et al., 2016; Zhang et al., 2023). The WME estimation results are preferred when there is only heterogeneity but no pleiotropy. The weighted mode approach also weighted the causal effect first and then took its mode as the final causal effect estimate. When causal effect estimates of similar individual SNPs were derived from valid SNPs, the estimates remained consistent even when the SNPs themselves were invalid (Wu et al., 2023). The simple mode approach was the unweighted mode of the causal effect estimate, which had less bias than other models but less accuracy, because it could reduce bias (Hartwig et al., 2017; Milne et al., 2017). We followed the three major principles for selecting MR methods as given below: (1) In the absence of pleiotropy and heterogeneity, the IVW estimation results are preferred; (2) the WME estimation results are preferred when there is only heterogeneity but no pleiotropy, and in addition, the IVW random effects model can be used too; (3) the MR-Egger estimation results are preferred when there is pleiotropy (Burgess and Thompson, 2017). When it comes to the case of genetic variables that violate the assumption of pleiotropy, the weighted mode approach can be employed (Wu et al., 2023). Although the simple mode approach may not be statistically as powerful as IVW, it can provide robustness against pleiotropy effects (Milne et al., 2017).

### Sensitivity analysis

To evaluate the possible influence of pleiotropy and heterogeneity among IVs on MR results, we employed several methods for sensitivity analysis to ensure the obtained robustness of the results. We applied the MR-Egger regression to assess horizontal pleiotropy. When the intercept term was not equal to zero and the *p-*value was less than 0.05, gene pleiotropy was considered to exist (Burgess and Thompson, 2017). In addition, the MR pleiotropy residual sum and outlier (MR-PRESSO) method was performed to judge instances of horizontal pleiotropy and remove significant outliers (Verbanck et al., 2018). Moreover, Cochran’s *Q* test and funnel plots were applied to judge the heterogeneity among the selected IVs (Greco et al., 2015). Furthermore, in order to determine whether a single SNP could have an impact on the main causal relationship, the “leave-one-out” analysis was conducted by sequentially eliminating each SNP. We used the “TwoSampleMR” and “MRPRESSO” packages in R v4.3.1 for all statistical analyses (Hemani et al., 2018).

## Results

### Selection of instrumental variables

According to the selection criteria of IVs, we first identified 7,089 unique SNPs for 119 genera as IVs from the MiBioGen Consortium (whole-genome significance threshold *p* < 1 × 10^−5^). Then, the aforementioned quality control processes were carried out, and 1,531 unique SNPs were finally identified for the MR analysis. Additionally, the *F*-statistics of all the screened IVs ranged between 14.58 and 88.42 (*F*-statistic >10), suggesting no bias evidence of weak instrument. The important data of the IVs mentioned above, including *F*-statistic, *R*^2^, *p*-value, SE, beta, the effect allele, and the other allele, are presented in Supplementary Table S1.

### Causal effects of gut microbiota on synovitis and tenosynovitis

**Figure 3:**
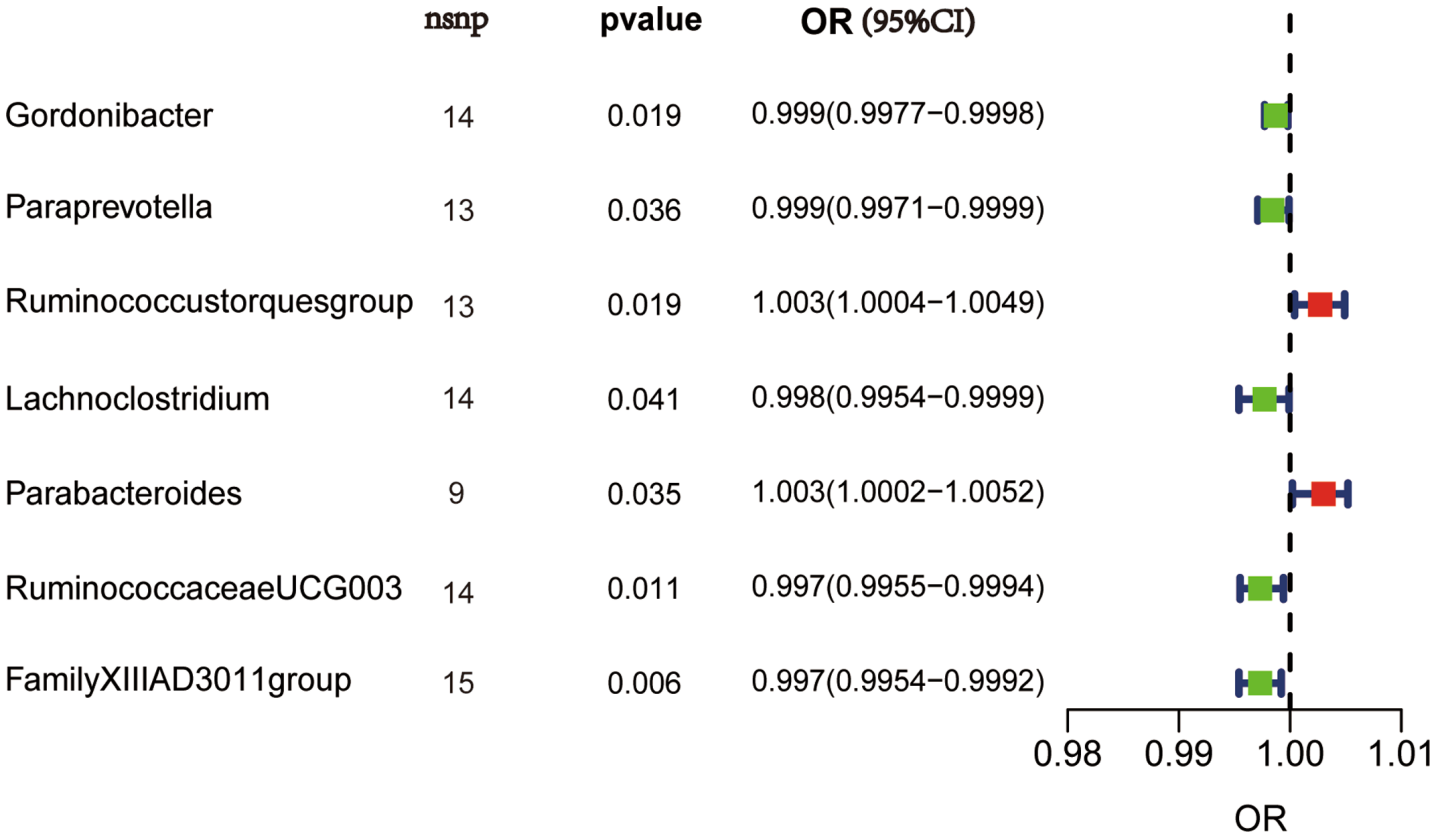
IVW results in forest plot for the causal effects of gut microbiota on synovitis and tenosynovitis. Nsnp, number of SNPs; OR, odds ratio; CI, confidence interval.

We performed MR analysis using the aforementioned five statistical models and used odds ratio (OR) to represent the degree and trend of association between gut microbiota with synovitis and tenosynovitis. Employing the IVW approach, seven bacterial genera that have a potential causal relationship with synovitis and tenosynovitis were identified. As shown in Figure 3 and Table 1, the IVW technique demonstrated that genetically predicted five genera including *Gordonibacter* [OR = 0.999, 95% confidence interval (CI): (0.9977, 0.9998), *p* = 0.019], *Paraprevotella* [OR = 0.999, 95% CI: (0.9971, 0.9999), *p* = 0.036], *Lachnoclostridium* [OR = 0.998, 95% CI: (0.9954, 0.9999), *p* = 0.041], *RuminococcaceaeUCG003* [OR = 0.997, 95% CI: (0.9955, 0.9994), *p* = 0.011], and *FamilyXIIIAD3011group* [OR = 0.997, 95% CI: (0.9954, 0.9992), *p* = 0.006] were negatively correlated with the risk of synovitis and tenosynovitis, while two other genera including *Ruminococcustorquesgroup* [OR = 1.003, 95% CI: (1.0004, 1.0049), *p* = 0.019] and *Parabacteroides* [OR = 1.003, 95% CI: (1.0002, 1.0052), *p* = 0.035] were positively associated with synovitis and tenosynovitis risk. Detailed information is provided in Supplementary Tables S2, S3.

**Table 1:** MR estimation of the relationship between gut microbiota with synovitis and tenosynovitis (*p* < 1 × 10^−5^).

Microbiota	SNPs	Methods	Beta	OR (95% CI)	*p*-value
Gordonibacter	14	MR-Egger	−0.0031	0.997 (0.9928, 1.0011)	0.170
		Weighted median	−0.0009	0.999 (0.9976, 1.0005)	0.212
		IVW	−0.0012	0.999 (0.9977, 0.9998)	0.019
		Simple mode	−0.0009	0.999 (0.9965, 1.0016)	0.470
		Weighted mode	−0.0009	0.999 (0.9965, 1.0018)	0.532
Paraprevotella	13	MR-Egger	−0.0016	0.998 (0.9939, 1.0029)	0.493
		Weighted median	−0.0012	0.999 (0.9970, 1.0007)	0.233
		IVW	−0.0015	0.999 (0.9971, 0.9999)	0.036
		Simple mode	−0.0001	0.999 (0.9969, 1.0029)	0.940
		Weighted mode	−0.0004	0.999 (0.9969, 1.0023)	0.782
Ruminococcustorquesgroup	13	MR-Egger	0.0035	1.004 (0.9967, 1.0104)	0.335
		Weighted median	0.0037	1.004 (1.0007, 1.0067)	0.017
		IVW	0.0026	1.003 (1.0004, 1.0049)	0.019
		Simple mode	0.0043	1.004 (0.9989, 1.0098)	0.146
		Weighted mode	0.0041	1.004 (0.9994, 1.0090)	0.116
Lachnoclostridium	14	MR-Egger	−0.0016	0.998 (0.9902, 1.0067)	0.716
		Weighted median	−0.0015	0.998 (0.9952, 1.0017)	0.359
		IVW	−0.0024	0.998 (0.9954, 0.9999)	0.041
		Simple mode	−0.0009	0.999 (0.9933, 1.0050)	0.779
		Weighted mode	−0.0007	0.999 (0.9938, 1.0049)	0.809
Parabacteroides	9	MR-Egger	0.0020	1.002 (0.9951, 1.0090)	0.586
		Weighted median	0.0030	1.003 (0.9998, 1.0063)	0.066
		IVW	0.0027	1.003 (1.0002, 1.0052)	0.035
		Simple mode	0.0041	1.004 (0.9989, 1.0093)	0.164
		Weighted mode	0.0041	1.004 (0.9995, 1.0088)	0.121
RuminococcaceaeUCG003	14	MR-Egger	−0.0013	0.999 (0.9921, 1.0055)	0.721
		Weighted median	−0.0031	0.997 (0.9944, 0.9995)	0.018
		IVW	−0.0026	0.997 (0.9955, 0.9994)	0.011
		Simple mode	−0.0042	0.996 (0.9916, 1.0001)	0.081
		Weighted mode	−0.0042	0.996 (0.9915, 1.0002)	0.082
FamilyXIIIAD3011group	15	MR-Egger	−0.0039	0.996 (0.9869, 1.0054)	0.426
		Weighted median	−0.0027	0.997 (0.9947, 0.9999)	0.048
		IVW	−0.0027	0.997 (0.9954, 0.9992)	0.006
		Simple mode	−0.0027	0.997 (0.9929, 1.0017)	0.242
		Weighted mode	−0.0028	0.997 (0.9928, 1.0017)	0.246

### Sensitivity analysis

**Figure 4:**
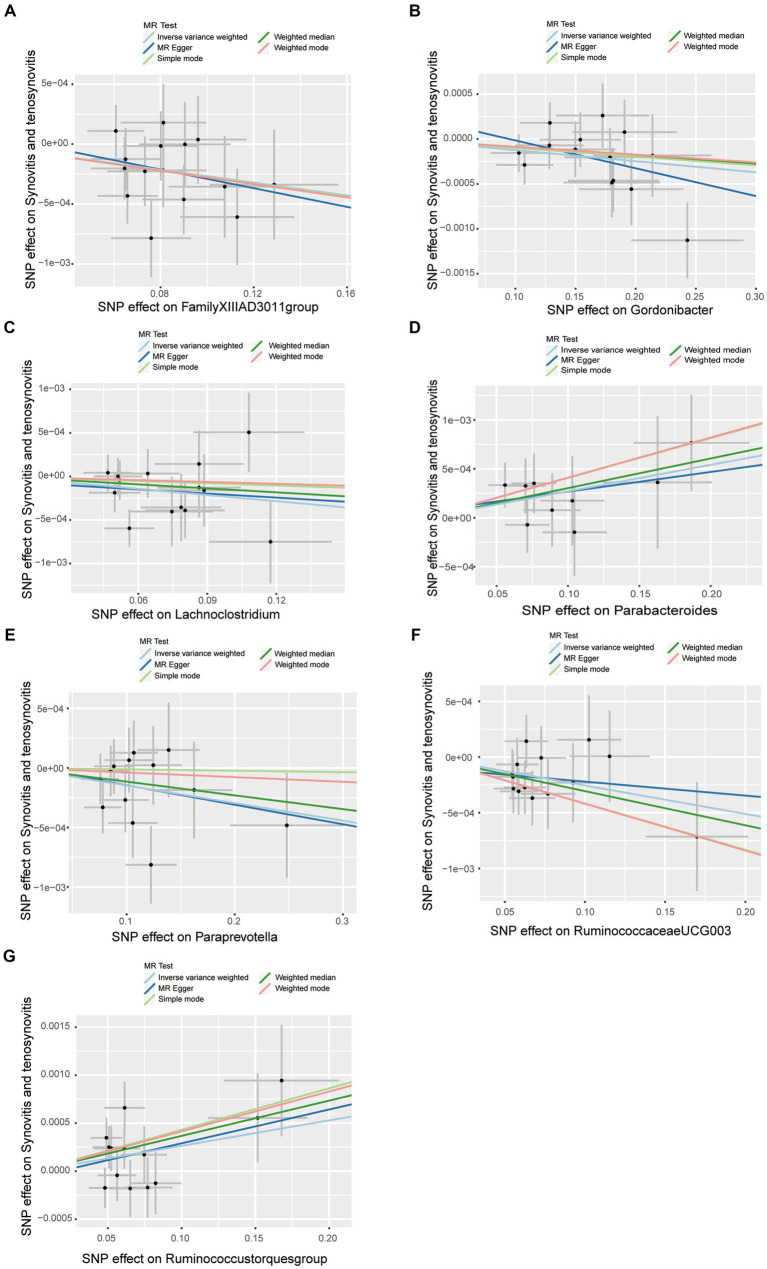
Scatter plots of causal estimates of gut microbiota on synovitis and tenosynovitis. **(A)** Genus *FamilyXIIIAD3011group*; **(B)** Genus *Gordonibacter*; **(C)** Genus *Lachnoclostridium*; **(D)** Genus *Parabacteroides*; **(E)** Genus *Paraprevotella*; **(F)** Genus *RuminococcaceaeUCG003*; and **(G)** Genus *Ruminococcustorquesgroup*. The slope of each line corresponds to the estimated MR effect in different models, including MR-Egger, weighted median, IVW, simple mode, and weighted mode. The Y-axis and X-axis represent the impact of IVs on the outcome and exposure, respectively, and the horizontal and vertical lines represent the 95% CI for IVs. MR Test, statistical analysis methods.

**Figure 5:**
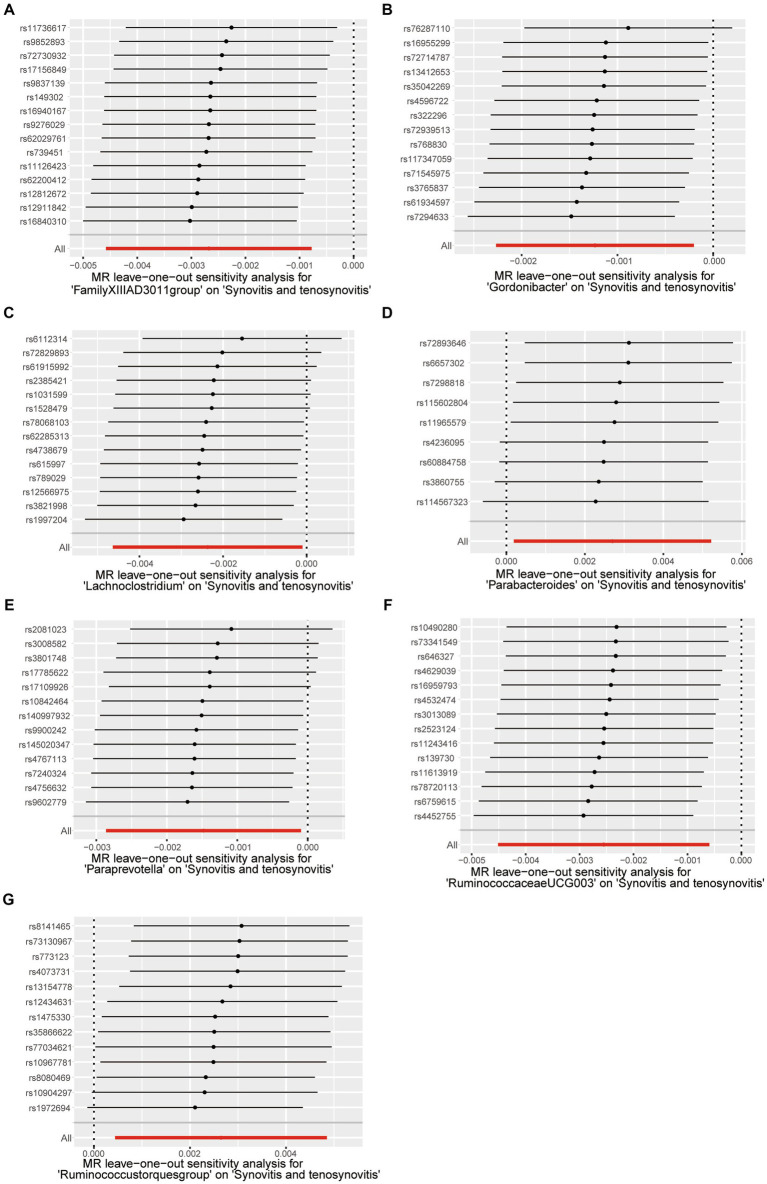
Leave-one-out test causal estimates of gut microbiota on synovitis and tenosynovitis. **(A)** Genus *FamilyXIIIAD3011group*; **(B)** Genus *Gordonibacter*; **(C)** Genus *Lachnoclostridium*; **(D)** Genus *Parabacteroides*; **(E)** Genus *Paraprevotella*; **(F)** Genus *RuminococcaceaeUCG003*; and **(G)** Genus *Ruminococcustorquesgroup*. Calculate the MR results of the remaining IVs after eliminating each SNP in turn. The plots of leave-one-out are employed to visualize the IVW overall estimate (red horizontal line) and the estimate after removing each IV (black horizontal line).

A series of comprehensive sensitivity analyses were carried out, in order to ensure the obtained robustness and reliability of the results (Supplementary Table S4). As shown in Table 2, the MR-Egger regression intercept term results suggested that the selected IVs did not exhibit horizontal pleiotropy (*p* > 0.05); besides, the MR-PRESSO global test results showed that no outliers were found. Meanwhile, Cochran’s *Q* test results indicated that there was no significant heterogeneity among these IVs (*p* > 0.05), and the funnel plots displayed general symmetry, which also showed that there was almost no heterogeneity (Supplementary Figure S1). Moreover, we provided detailed scatter plots for the above-mentioned five MR methods in Figure 4. After analyzing using the leave-one-out analysis, we found that no single SNP exerted a large impact on the total estimations, which were referred to in Figure 5.

**Table 2:** Evaluation of horizontal pleiotropy and heterogeneity using different methods.

Microbiota	Horizontal pleiotropy		Heterogeneity	
	MR-Egger intercept *p*	MR-PRESSO global test *p*	MR-Egger Cochran’s Q *p*	IVW Cochran’s Q *p*
Gordonibacter	0.382	0.614	0.590	0.601
Paraprevotella	0.943	0.660	0.557	0.641
Ruminococcustorquesgroup	0.796	0.466	0.341	0.415
Lachnoclostridium	0.847	0.547	0.475	0.555
Parabacteroides	0.839	0.909	0.851	0.907
RuminococcaceaeUCG003	0.699	0.943	0.915	0.939
FamilyXIIIAD3011group	0.797	0.740	0.669	0.735

## Discussion

As far as we know, this study is the first two-sample MR analysis to elucidate the causal relationship of gut microbiota to synovitis and tenosynovitis by using publicly available genetic databases. Based on our study findings, seven kinds of gut microbiota had a potential causal relationship with the progression of synovitis and tenosynovitis, among which five genera including *Gordonibacter*, *Paraprevotella*, *Lachnoclostridium*, *RuminococcaceaeUCG003*, and *FamilyXIIIAD3011group* were negatively correlated with synovitis and tenosynovitis, while two genera including *Ruminococcustorquesgroup* and *Parabacteroides* were positively correlated with synovitis and tenosynovitis. Moreover, pleiotropy or heterogeneity was not present in our study, and sensitivity analysis suggested that our results are credible. This MR analysis strengthened the comprehension of the pathological impact of gut microbiota on the occurrence and development of synovitis and tenosynovitis and provided a new angle of view for the prevention, diagnosis, and treatment of synovitis and tenosynovitis. Remarkably, our study emphasizes that changes in both the abundance and diversity of the gut microbiota may be a factor contributing to the onset of synovitis and tenosynovitis. Consequently, changing the abundance and diversity of these gut microbiota is expected to become a measure to prevent and treat synovitis and tenosynovitis, and its application prospects are gradually emerging. However, the need for further extensive research in this regard must be emphasized.

There are trillions of microorganisms colonizing the human gut to form the gut microbiota, which is approximately 10 times the number of human cells, giving the host multiple functions (Ley et al., 2006; Rooney et al., 2021). Synovitis and tenosynovitis are common in joint diseases, such as RA, OA, and so on (Dakkak et al., 2020; Lin et al., 2020). The interaction between gut microbiota and joint diseases is often described in terms of the “gut–joint axis” (Zaiss et al., 2021). As part of the gut–joint axis, dysbiosis of gut microbiota can cause immune system disorders and a sustained immune inflammatory response, leading to the occurrence and development of synovitis and tenosynovitis (Romero-Figueroa et al., 2023). To date, there has been accumulating evidence that the gut microbiota is involved in human joint diseases through multiple pathways (Yang and Cong, 2021). Some scholars have found that compared to healthy controls, *Lachnoclostridium* was significantly lower in the RA (Liu et al., 2020). In the treatment of OA, *Lachnoclostridium* in the electroacupuncture group was statistically higher than baseline, and electroacupuncture could treat OA by regulating the gut microbiota (Wang et al., 2021a). In addition, some researchers have also explored that a positive correlation exists between *Parabacteroides* and erythrocyte sedimentation rate (ESR) in RA patients (Sun et al., 2019). Moreover, the imbalance of *Parabacteroides* and *Gordonibacter* was closely related to the onset of OA (Ramasamy et al., 2021), and *Parabacteroides* could be used as an early diagnostic biomarker for OA (Wang et al., 2021b). Previous studies had also shown that the abundance of *Parabacteroides* in mice with adjuvant-induced arthritis (AIA) was significantly increased, and its level was decreased after treatment, indicating a positive correlation between the occurrence of AIA and *Parabacteroides* (Hu et al., 2023). Given the association of RA and OA with synovitis and tenosynovitis, the above research results can also support some of the conclusions of our MR analysis.

Within our investigation, we also found that genera *RuminococcaceaeUCG003* and *FamilyXIIIAD3011group* were negatively related to synovitis and tenosynovitis, while the genus *Ruminococcustorquesgroup* was positively associated with them. It is worth noting that so far, there have been no clinical studies documenting alterations in the diversity or abundance of these bacteria in patients with synovitis and tenosynovitis or related diseases. Our research tentatively posits that these changes in bacteria levels might have an impact on the development of synovitis and tenosynovitis. According to our research outcomes, the management of synovitis and tenosynovitis can be achieved by regulating the diversity and abundance of the aforementioned gut microbiota, but further exploration of these aspects is needed.

Our study had the following advantages. First, the MR method was utilized to infer the association of gut microbiota with synovitis and tenosynovitis, which could reduce the distractions of false causal relationships and confounding factors. Second, the use of publicly available comprehensive GWAS data to obtain genetic variations in gut microbiota ensured both high sample sizes and the reliability of the analytical tools. Third, because we analyzed the causal role of the gut microbiota in synovitis and tenosynovitis at the genus level, it could pinpoint particular candidate bacteria for functional studies and clinical practice in the future. Finally, multiple statistical models, various sensitivity analysis approaches, and stringent quality control procedures ensured the robustness and reliability of causal effect estimation.

Nevertheless, our study also presented the following several limitations. First, the employed GWAS data only involved subjects of European ancestry in this study, and it was unclear whether our findings were applicable to other races. Moreover, in the GWAS database, the gut microbiota data of other ethnic groups were limited, and as there may be differences in gut microbiota composition among different ethnic groups, this discrepancy may affect our findings. Second, we only analyzed bacterial taxa at the genus level, which was the lowest classification level in the original GWAS data, thus limiting the capacity to reveal the causality of gut microbiota with synovitis and tenosynovitis at more specific species levels. Therefore, further investigation needs analysis at higher levels of taxonomy (e.g., phylum, class, and order), and we need further studies to get the GWAS statistical data of genetic variation for bacterial species. Third, owing to the lack of basic demographics of the original research subjects (e.g., gender and age), these factors were not taken into account in the data analysis, and further research is needed to determine whether they have an impact on the results. For our findings, it is necessary to consider whether there would be any difference when applied to different age or gender groups. Fourthly, although our GWAS data utilized in this study derived from the biggest and most complete macro-genome sequencing study currently available, aggregated data on other gut microbiota are needed in the future to more fully evaluate the causal association of gut microbiota with synovitis and tenosynovitis. Furthermore, the GWAS of gut microbiota are at their early stage in terms of sample size, and the loci linked with synovitis and tenosynovitis are relatively few. In order to perform sensitivity analysis and test at a variety of significance levels, more genetic variants need to be included as IVs. Finally, due to the complex and diverse gut microbiota, our study did not consider whether the complex interrelationships among different types of microbial communities would affect synovitis and tenosynovitis in the case of imbalanced gut microbiota. Therefore, further studies are necessitated to investigate the intricate interactions between these factors.

It should be noted that despite the research has limitations, we remain optimistic about the academic implications of this study. Although our investigation reveals a seemingly reasonable causal relationship between gut microbiota with synovitis and tenosynovitis, it should be acknowledged that MR study is a hypothesis-based method and there is a lack of direct mechanistic studies to confirm our findings. Therefore, subsequent clinical and experimental studies are essential for establishing the causal connection between gut microbiota and specific diseases. Future research should aim to validate our current findings by conducting clinical studies with diverse populations and larger sample sizes or conducting further laboratory-based research, so as to establish the robustness of the obtained results. Further study methods include but are not limited to: (1) implementing appropriate prospective cohort studies; (2) studies about transplanting the microbial species of interest into germ-free animals; and (3) studies on microbiome analysis of patient fecal samples by microbial sequencing. All the methods listed above have their advantages and disadvantages, but they will help us to have a better understanding of the gut microbiota’s role in synovitis and tenosynovitis.

To sum up, our study is the inaugural endeavor to use MR analysis to investigate the causal effects of gut microbiota on synovitis and tenosynovitis. Specific groups of bacteria that may affect synovitis and tenosynovitis were identified using publicly available GWAS summary statistical data. Our results suggest that gut microbiota plays a bidirectional role in synovitis and tenosynovitis. Five genera, *Lachnoclostridium*, *Gordonibacter*, *Paraprevotella*, *RuminococcaceaeUCG003*, *and FamilyXIIIAD3011group*, were related to a reduced risk of synovitis and tenosynovitis, whereas two genera including *Ruminococcustorquesgroup* and *Parabacteroides* were related to an increased risk of synovitis and tenosynovitis. This study provides higher reliability results than conventional observational studies that are susceptible to confounding factors. The gut microbiota-linked SNPs used in our study were derived from the most extensive GWAS survey conducted to date, confirming the IVs robustness incorporated in this study. The combination of the large sample size and the application of different sensitivity analyses ensures the validity and resilience of the research results. Our results provide valuable directions and insights into the treatment and prevention of synovitis and tenosynovitis for further studies. However, it must be acknowledged that direct mechanistic studies are lacking to confirm our findings. Future studies should focus on more diversified and comprehensive investigations to further deepen our understanding of the complex connection between diseases and gut microbiota.

## Conclusion

In summary, employing our two-sample MR analysis, this study systematically revealed the causality of gut microbiota with synovitis and tenosynovitis for the first time and provided comprehensive screening data for the relationship between the two. Our research results indicated that seven bacterial genera might have a causal effect on synovitis and tenosynovitis, with five being negatively correlated and the other two positively correlated. These findings highlighted that gut microbiota have played a very important role in synovitis and tenosynovitis pathogenesis, which would provide new clinical ideas for the treatment and prevention of synovitis and tenosynovitis by targeting specific gut microbiota. However, more mechanistic research and clinical exploration are required in the future to confirm these findings.

## Data availability statement

The original contributions presented in the study are included in the article/Supplementary material, further inquiries can be directed to the corresponding author.

## Author contributions

XTY: Methodology, Project administration, Writing – original draft, Writing – review & editing. SCZ: Data curation, Writing – original draft. ZKT: Validation, Writing – original draft. JX: Data curation, Writing – original draft. QPL: Validation, Writing – review & editing.

## Footnotes

^1^ https://mibiogen.gcc.rug.nl/

^2^ https://gwas.mrcieu.ac.uk/
