# Supplementary material for: Causal associations between gut microbiota and synovitis–tenosynovitis: a two-sample Mendelian randomization study
Source: Front Microbiol. 2024 Apr 30;15:1355725. doi: 10.3389/fmicb.2024.1355725 (PMC11091245; doi:10.3389/fmicb.2024.1355725)
Supplement: Supplementary file 5 [file Data_Sheet_1.DOCX]

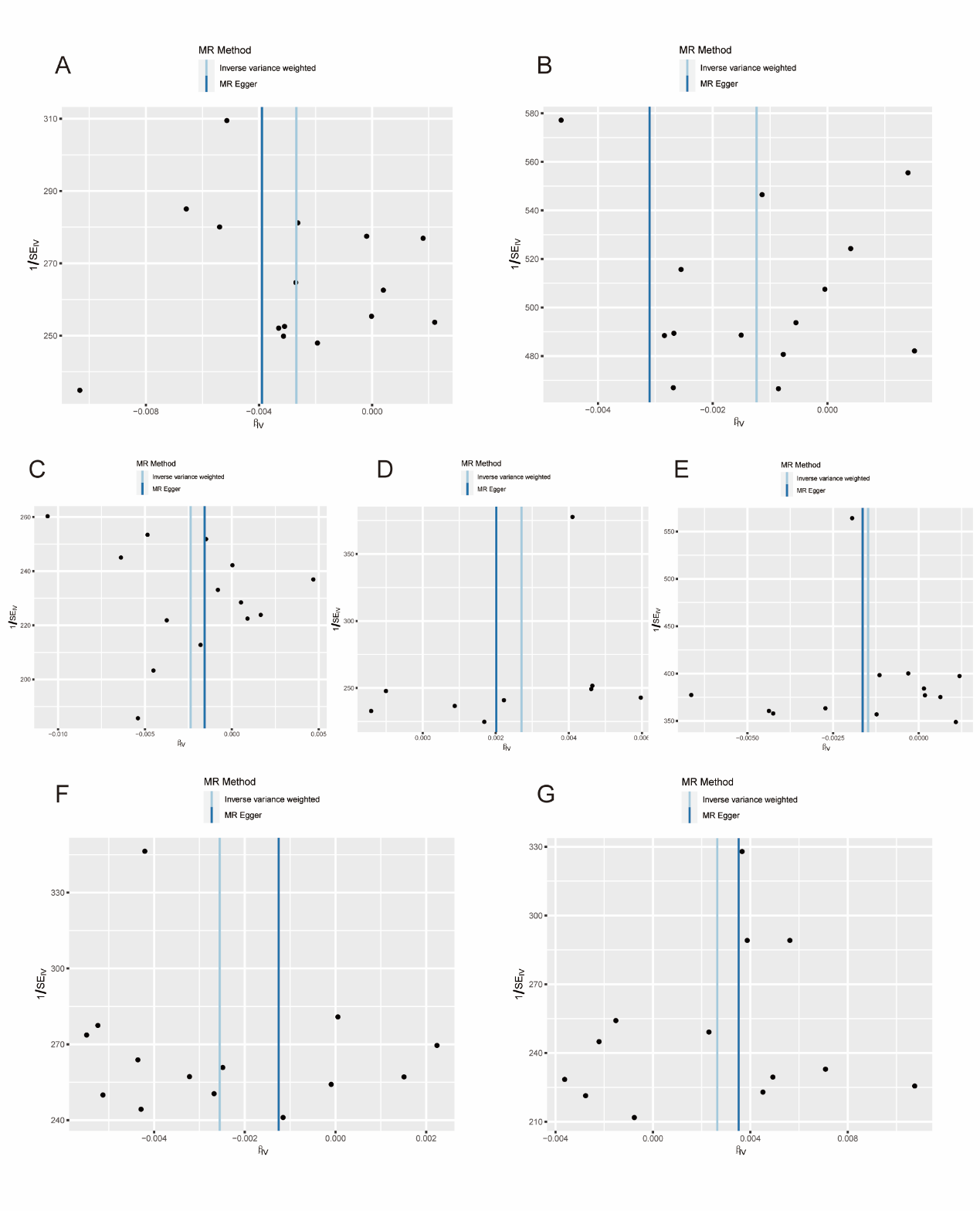


SUPPLEMENTATY FIGURE S1 | Funnel plots for MR analyses of the causal effects of gut microbiota on synovitis and tenosynovitis. (A) Genus *FamilyXIIIAD3011group*; (B) Genus *Gordonibacter*; (C) Genus *Lachnoclostridium*; (D) Genus *Parabacteroides*; (E) Genus *Paraprevotella*; (F) Genus *RuminococcaceaeUCG003*; (G) Genus *Ruminococcustorquesgroup*. Funnel plots are scatter plots in which the estimated causal effects of IVs are plotted according to their precision. The Y-axis represents the estimated causal effects of each genetic variation, while the X-axis represents the accuracy of causal estimation. In the unbiased case where there is no heterogeneity, the scatter points on the funnel plot should show a symmetrical distribution similar to the shape of an inverted funnel.
